# Synthesis of a Magnetic Carnation-like Hydroxyapatite/Basic Calcium Carbonate Nanocomposite and Its Adsorption Behaviors for Lead Ions in Water

**DOI:** 10.3390/molecules27175565

**Published:** 2022-08-29

**Authors:** Haifeng Guo, Siru Hu, Zongli Wang, Yutong Li, Xinshuang Guo, Ziling He, Wenbin Wang, Jun Feng, Kangyun Yang, Hong Zheng

**Affiliations:** 1 Engineering & Technology Research Center for Environmental Protection Materials and Equipment of Jiangxi Province, College of Materials and Chemical Engineering, Pingxiang University, Pingxiang 337055, China; 2 School of Statistics and Data Science, Nankai University, Tianjin 300071, China

**Keywords:** hydroxyapatite, basic calcium carbonate, hierarchical nanocomposite, adsorption, heavy metal ions

## Abstract

Calcium-enriched compounds have great potential in the treatment of heavy-metal contaminated wastewater. Preparing stable basic calcium carbonate (BCC), which is a calcium-enriched compound, and applying it in practice is a great challenge. This work investigated the formation process of hierarchical hydroxyapatite (HAP)/BCC nanocomposites and their adsorption behaviors regarding lead ions (Pb^2+^). The morphology of the HAP/BCC nanocomposite was controlled by the addition of monododecyl phosphate (MDP). The carnation-like HAP/BCC nanocomposite was achieved with the addition of 30 g of MDP. The carnation-like HAP/BCC nanocomposite had a high Pb^2+^ adsorption capacity of 860 mg g^−1^. The pseudo-second-order and Freundlich model simulation results indicated that the adsorptions of Pb^2+^ on the nanocomposites belonged to the chemisorption and multilayer adsorption processes. The main effective adsorption components for the nanocomposites were calcium-enriched HAP and BCC. Through the Ca^2+^ ions exchanging with Pb^2+^, the HAP and BCC phases were converted to hydroxyl-pyromorphite (Pb-HAP) and hydrocerussite (Pb_3_(CO_3_)_2_(OH)_2_), respectively. The carnation-like HAP/BCC nanocomposite has great potential in the treatment of heavy metal ions. This facile method provides a new method for preparing a stable HAP/BCC nanocomposite and applying it in practice.

## 1. Introduction

The treatment of wastewater polluted by heavy metal ions has become one of the most onerous challenges for the sustainable development of human society because of their detrimental effects on the environment and human health [1,2,3,4]. As one of the major heavy metals in the natural environment, lead (Pb) is considered a serious threat to the ecological environment and human health due to its high toxicity and persistent characteristics [5]. Lead contaminants enter the food chain mainly through crop irrigation and soil contamination [6]. According to the World Health Organization (WHO), inorganic lead compounds have been placed in Group 2A (probable carcinogens) and are described as probable human carcinogens, with a recommended maximum concentration of 10 μg L^−^^1^ in drinking water [7]. Exceedingly high lead exposure will cause bone degeneration, liver damage, lung insufficiency, hypertension, and renal dysfunction in humans [8]. Because of the high biological activity and its adverse influence on human health, even at low concentrations, Pb^2+^ ions should be removed from aqueous solutions [9,10].

Calcium contained compounds, such as calcium alginate (C_18_H_24_CaO_19_) [11], calcium carbonate (CaCO_3_) [12], calcium silicate hydrate (xCaO·SiO_2_·yH_2_O) [13,14], and hydroxyapatite (Ca_10_(PO_4_)_6_(OH)_2_) [15,16,17], are usually used as solid-phase extraction adsorbents for heavy metal ions. For these adsorbents, calcium ions play a key role in the solid-phase extraction-adsorption process by replacing heavy metal ions of aqueous solutions. Since these solid-phase extraction adsorbents come from calcium-enriched compounds, they can provide more calcium ions to replace heavy metal ions of aqueous solutions, and then they have higher adsorption efficiency for heavy metals [13,14,16]. Therefore, as a calcium-enriched compound, hydroxyapatite (HAP) contains ten calcium atoms in a unit cell and has excellent adsorption property for various heavy metal ions [15,16,17,18,19]. As a relevant phase of calcium carbonate, basic calcium carbonate (BCC) (Ca_3_(CO_3_)_2_(OH)_2_·H_2_O), which has three calcium atoms in a unit cell, is also a calcium-enriched compound. However, there are only a few studies regarding the synthesis of BCC because it is a metastable transition phase generated as a precursor when precipitated calcium carbonate is synthesized by a carbonation process, and it is not easily synthesized [20,21,22]. To our knowledge, there are no reports concerning the adsorption of heavy metal ions in regards to BCC due to the fact that BCC is unstable, and making it stable for industrial use is a great challenge [21,22]. Moreover, steel slag is a hazardous solid waste derived from the steel-making process, accounting for approximately 15–20% of the total crude steel output [23,24,25]. Most of steel slags are landfilled or piled up, occupying precious land resources and causing environmental risk of leaching heavy metal ions into the environment [26,27,28,29]. Tighter legislation and increasingly strict environmental regulations provide incentives to develop alternative viable reuse and recycling options to turn hazardous steel slags into environmentally friendly materials [29,30].

In this study, a magnetic carnation-like HAP/BCC nanocomposite was first synthesized and then successfully utilized as the adsorbent for Pb^2+^ in an aqueous solution. The morphology of the magnetic nanocomposite was controlled by the addition of monododecyl phosphate (MDP). Solid waste steel slag was used as the source of magnetism and partial Ca^2+^. The adsorption behaviors of Pb^2+^ upon the magnetic nanocomposites were investigated according to the adsorption kinetics and isotherms. The BCC phase, complexed with HAP in the obtained nanocomposite, was stable and greatly improved the adsorption property of Pb^2+^. The magnetic carnation-like HAP/BCC nanocomposite had a high Pb^2+^ adsorption capacity of 860 mg g^−^^1^ and good magnetic recovery efficiency, giving it great potential for the treatment of heavy-metal contaminated wastewater and soil. More importantly, this facile method provides a prospective strategy for preparing a stable HAP/BCC nanocomposite and applying it in practice.

## 2. Materials and Methods

### 2.1. Chemicals

The following chemicals were used in the study: calcium nitrate tetrahydrate (Ca(NO_3_)_2_·4H_2_O, Chemical Purity, Sinopharm Chemical Co., Ltd., Shanghai, China), diammonium hydrogen phosphate ((NH_4_)_2_HPO_4_, Chemical Purity, Sinopharm Chemical Co., Ltd., Shanghai, China), ammonia (NH_3_·H_2_O, Commercial Purity, Xilong Science Co., Ltd., Shantou, China), monododecyl phosphate (Ester, C_12_H_27_OPO_3_, Commercial Purity, Jiangsu Haian petrochemical plant, Haian, China), ethanol (C₂H₆O, Chemical Purity, Sinopharm Chemical Co., Ltd., Shanghai, China), and lead nitrate (Pb(NO_3_)_2_, Analytical purity, Sigma-Aldrich (St. Louis, MO, USA)). Steel slag (Jiangxi PXSTEEL industrial Co., Ltd., Pingxiang, China) was used as the source of magnetism and partial Ca^2+^ of the nanocomposite. After being processed under ball milling for 24 h, the steel slag was put through a 320-mesh sieve and then further selected using a neodymium magnet with a remanence of about 1.2 T. The final selected powder was used as the raw steel slag powder (sample S0).

### 2.2. Preparation of Magnetic HAP/BCC Nanocomposites

Varying amounts (0 g, 10 g, and 30 g, respectively) of MDP was initially dissolved in a solvent mixture composed of 45 mL water and 10 mL ethanol. After being stirred and refluxed at 60 °C for 1 h, the MDP solution was mixed with 20 g of raw steel slag powder. With additional mechanical stirring and refluxing at 60 °C for 1 h, a calcium nitrate tetrahydrate aqueous solution (0.167 mol of Ca(NO_3_)_2_·4H_2_O dissolved in 35 mL water) was added to the mixture. The mixture underwent an additional stirring and refluxing process at 60 °C for 1 h. Subsequently, diammonium hydrogen phosphate aqueous solution (0.1 mol of (NH_4_)_2_HPO_4_ dissolved in 20 mL water) was added into the above mixture solution until a Ca/P mol ratio of 1.67 was reached, and then the pH value of the mixture solution was adjusted to 13 by adding aqueous ammonia. Then, the final mixture was continuously stirred and refluxed at 60 °C for 48 h. The obtained products were centrifuged and washed with water and ethanol three times to obtain a gel. Finally, the gel was dried at 80 °C for 24 h and calcined at 300 °C for 3 h. The final three modified products were labeled as S1 for the addition of 0 g of MDP, S2 for the addition of 10 g of MDP, and S3 for the addition of 30 g MDP, respectively.

### 2.3. Batch Adsorption Experiments

Lead nitrate was used as the heavy metal ion source in the aqueous solution. The Pb^2+^ solution had a concentration of 100, 200, 300, 400, 500, 700, 1500, and 3000 mg L^−^^1^ (ppm), respectively. At room temperature (24 ± 1 °C), a 100 mL plastic tube containing 50 mL of heavy metal ion solution was added to 0.06 g of each sample (S0, S1, S2 and S3, respectively). After different periods (10 min, 20 min, 40 min, 1 h, 3 h, 5 h, 12 h, and 24 h) of oscillation in a gyro oscillator, the mixture was centrifuged at a speed of 5000 r min^−^^1^, and the upper liquid was removed for measurement. The concentrations of the heavy metal ion solutions before and after adsorption were measured separately. Three groups of parallel experiments were conducted, and the average values were obtained. The adsorption capacity of the heavy metal ions was calculated according to the concentration difference, the volume of the heavy metal ion solution, and the addition of different sample powders [13,14,16].

### 2.4. Characterization Methods

The raw steel slag and the obtained nanocomposite samples were characterized by X-ray diffraction (XRD, Bruker D8, Karlsruhe, Germany), transmission electron microscopy (TEM) (Philips, EM20, Eindhoven, The Netherlands), and scanning electron microscopy (SEM, Hitachi 8010, Tokyo, Japan), respectively. The Brunauer-Emmett-Teller (BET) specific surface area of all samples was analyzed using the nitrogen adsorption method (Quantachrome Corporation, Tristar 3020, Boynton Beach, FL, America). The pore size distribution was plotted using the Barrett-Joyner-Halenda (BJH) method. The mapping data of the energy dispersive spectroscopy (EDS) for the samples were tested by TEM QUANTAX EDS (Bruker, Karlsruhe, Germany). Atomic absorption spectrometry (PinAAcle 900H, PerKinElmer, Waltham, MA, America) was used to measure the concentration of the heavy metal ion solutions before and after adsorption. The magnetic characteristics were measured on a vibration sample magnetometer (VSM, Lake Shore 7410, Columbus, OH, America).

## 3. Results and Discussion

### 3.1. Characterization of Magnetic HAP/BCC Nanocomposites

**Figure 1:**
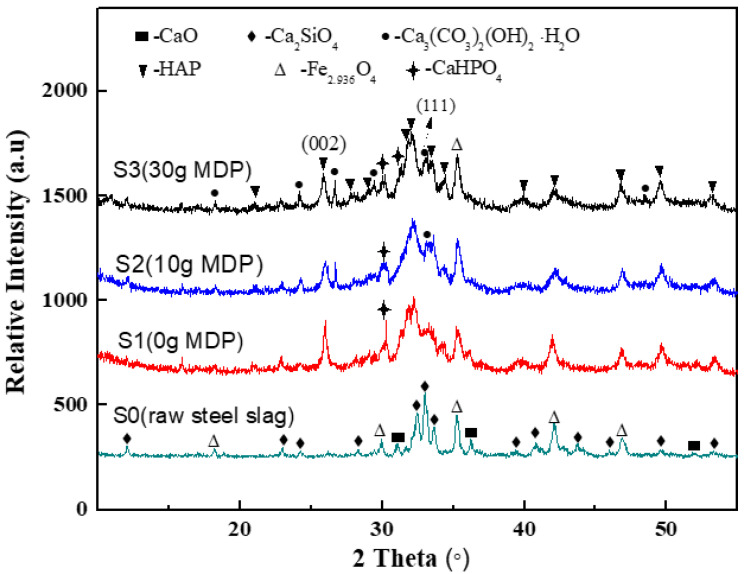
The XRD patterns of raw steel slag and the nanocomposite samples prepared with different additions of MDP.

Figure 1 presents the XRD patterns of raw steel slag and the magnetic HAP/BCC nanocomposite samples prepared with different additions of MDP. The raw steel slag (S0) mainly contained Ca_2_SiO_4_ (JCPDS No. 00-024-0037), CaO (JCPDS No. 96-900-6740), and Fe_2.936_O_4_ (JCPDS No. 01-086-1353). For sample S1, prepared without the addition of MDP, the main phases were HAP (JCPDS No. 96-900-2214) and Fe_2.936_O_4_ of steel slag, with a small amount of CaHPO_4_ (JCPDS No. 00-003-0423), whereas samples S2 (with 10 g MDP) and S3 (with 30 g MDP) mainly contained HAP, Fe_2.936_O_4_, and a new phase—BCC (Ca_3_(CO_3_)_2_(OH)_2_·H_2_O, JCPDS No.00-023-0107). Moreover, sample S3 had a higher number of BCC diffraction peaks than S2, indicating that S3 had a greater BCC content than S2. The BCC content of S3 was about 2.31 times that of S2, according to the EDS mapping data, as shown in Figures S2 and S3 in the Supplementary Material (Conversely the HAP content of S2 was about 1.65 times than that of S3). After the reactions, the CaO phase of the raw steel slag disappeared.

**Figure 2:**
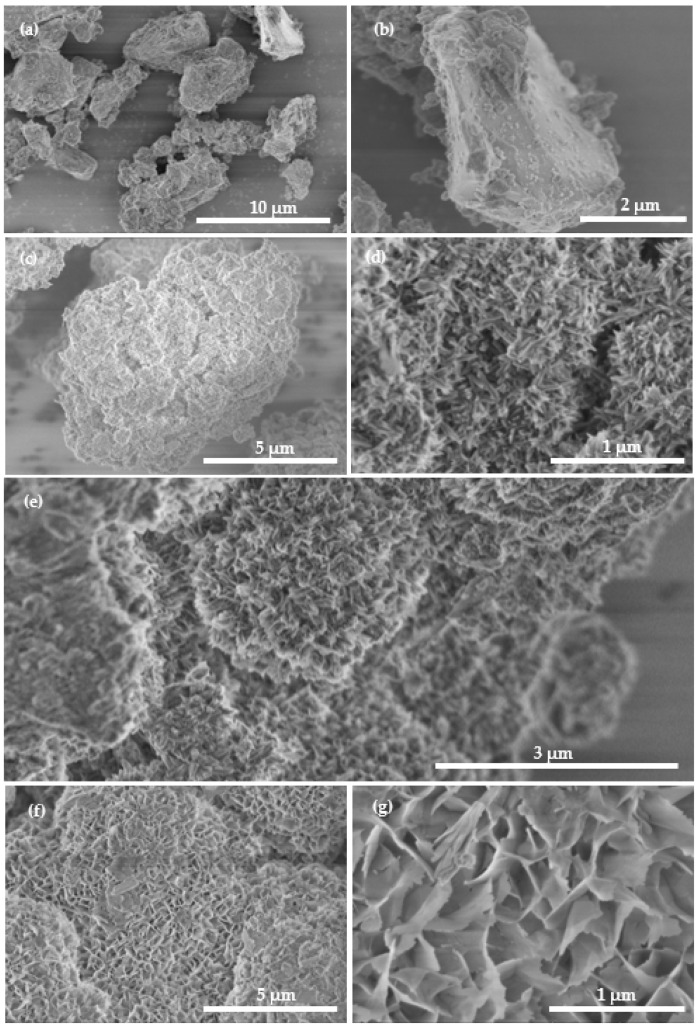
SEM micrographs of raw steel slag and the nanocomposite samples prepared with different additions of MDP: (**a**,**b**), raw steel slag (S0); (**c**,**d**), S1 (0 g MDP); (**e**), S2 (10 g MDP); (**f**,**g**), S3 (30 g MDP).

Figure 2 shows the SEM micrographs of raw steel slag and the magnetic HAP/BCC nanocomposite samples prepared with different additions of MDP. The raw steel slag (S0) had an irregular morphology with a particle size of 2–10 μm, and the particles had dense surfaces, without obvious pores or nanostructures. By contrast, the three nanocomposite samples (S1, S2, and S3) had loose surfaces, with outstanding pores and interesting hierarchical nanostructures: S1 demonstrated a lappa-like nanoflower morphology, which was composed of nanoneedles; S3 illustrated a carnation-like nanoflower morphology, which was composed of nanosheets; and S2 possessed both lappa-like and carnation-like morphologies. According to the results of XRD (Figure 1), the composition of nanoneedles for S1 was HAP, and that of the nanosheets for S3 belonged to the mixture of HAP and BCC.

**Figure 3:**
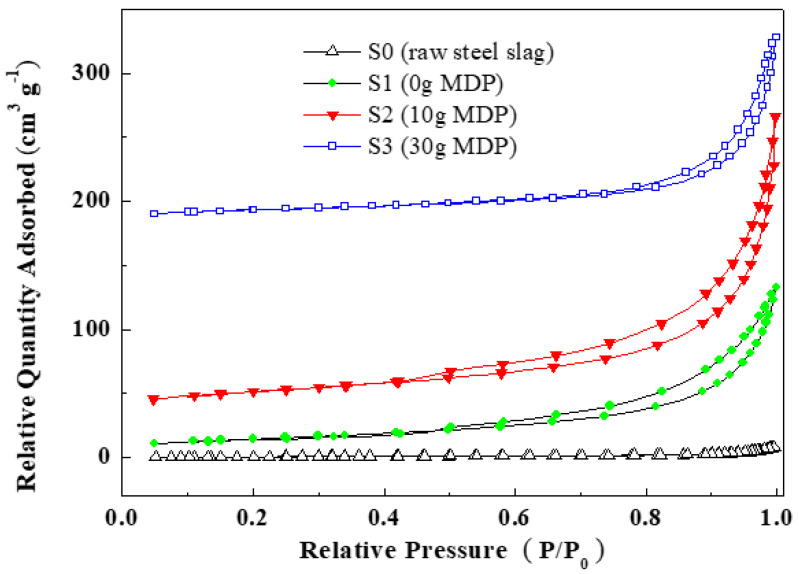
Nitrogen adsorption/desorption isotherm curves of raw steel slag and the nanocomposite samples prepared with different additions of MDP. The relative quantity adsorbed value was calculated for the standard temperature and pressure conditions.

**Figure 4:**
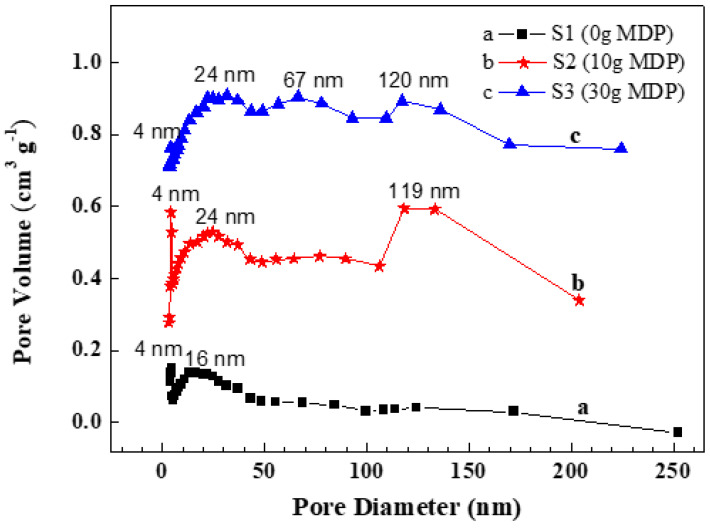
BJH pore size distribution of the nanocomposite samples prepared with different additions of MDP: (**a**) S1 (0 g MDP), (**b**) S2 (10 g MDP), and (**c**) S3 (30 g MDP).

In comparison with that of raw steel slag, the specific surface area of the nanocomposite was greatly improved, from 3.31 m^2^ g^−1^ (S0) to 52.24 m^2^ g^−1^ (S1), 78.94 m^2^ g^−1^ (S2), and 46.67 m^2^ g^−1^ (S3), respectively (Figure 3). Moreover, the nanoflowers provided the three nanocomposite samples many nanopores and multi pore size distributions, as shown in Figure 4. Sample S1 had a pore size of 4 nm and 16 nm, with a total pore volume of 0.221 cm^3^ g^−^^1^. S2 showed a pore size of 4 nm, 24 nm, and 120 nm, with a total pore volume of 0.355 cm^3^ g^−^^1^, and S3 had a pore diameter of 4 nm, 67 nm, and 120 nm, with a total pore volume of 0.225 cm^3^ g^−^^1^. S0 possessed a low total pore volume of 0.014 cm^3^ g^−^^1^, as shown in Figure S1 of the Supplementary Materials.

### 3.2. Adsorption Analysis

#### 3.2.1. Adsorption Kinetics

The kinetic adsorption data for Pb^2+^ on raw steel slag and the nanocomposite samples prepared with different additions of MDP were described by the pseudo-first-order and pseudo-second-order models. The pseudo-first-order model is presented as Equation (1):

q_t_ = q_e_(1 − e^−k1t^) (1)

The pseudo-second-order model equation is expressed as Equation (2):

q_t_ = k_2_q_e_^2^t/(1 + k_2_q_e_t) (2)

where q_e_ (mg g^−1^) is the amount of Pb^2+^ adsorbed at equilibrium time, q_t_ (mg g^−1^) is the amount of Pb^2+^ adsorbed by sample at time t (min), and k_1_ and k_2_ (mg·(g·min)^−1^) are the pseudo-first-order model rate and the pseudo-second-order rate, respectively.

**Figure 5:**
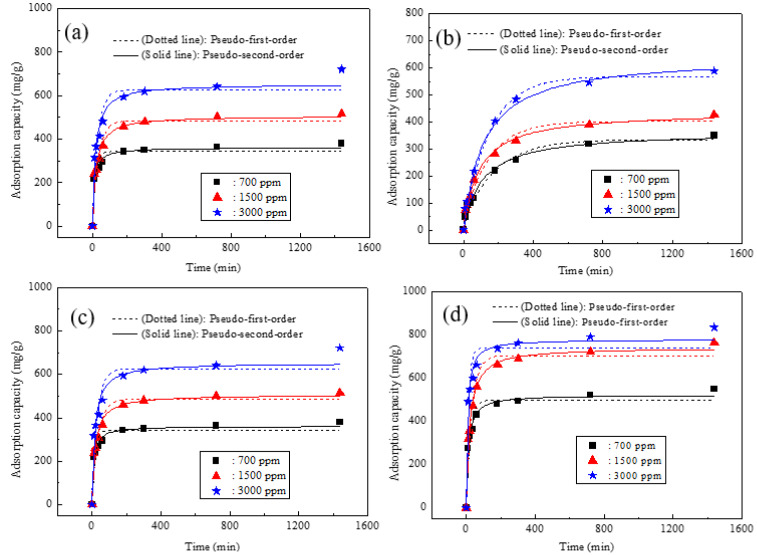
Kinetic adsorption curves with different concentrations of Pb^2+^ in raw steel slag and the nanocomposite samples prepared with different additions of MDP: (**a**) raw steel slag (S0), (**b**) S1 (0 g MDP), (**c**) S2 (10 g MDP), and (**d**) S3 (30 g MDP).

Figure 5 gives the kinetic adsorption curves for different concentrations of Pb^2+^ in raw steel slag and the nanocomposite samples prepared with different additions of MDP, and the fitting parameters are shown in Table S1 of the Supplementary Materials. For all the adsorption samples, the value of the correlation coefficient (R^2^) for the pseudo-second-order model was above 0.97 and was higher than that of the pseudo-first-order model, indicating that the pseudo-second-order model could better express the kinetic adsorption processes. This result implied that the main interaction of Pb^2+^ with all the adsorption samples was chemisorption, rather than mass transportation [9,31]. For all the four samples (S0, S1, S2, and S3), the absorption of Pb^2+^ increased rapidly within the first contact time of 60 min. While S0 and S1 achieved adsorption equilibrium after 720 min, S2 and S3 achieved adsorption equilibrium after 300 min, and all the four samples reached the maximum equilibrium adsorption capacity when the initial concentration of Pb^2+^ was 3000 ppm. The calculated maximum equilibrium adsorption capacities of S0, S1, S2, and S3 were 15.30 mg g^−^^1^, 641.5 mg g^−^^1^, 651.9 mg g^−^^1^, and 778.3 mg g^−1^, respectively (see Table S1 in the Supplementary Materials).

#### 3.2.2. Adsorption Isotherms

With the purpose of understanding the interaction mechanism of Pb^2+^ on the four absorbent samples, the Langmuir model and the Freundlich model were chosen to simulate the experiment data.

The Langmuir isotherm model is expressed as Equation (3):

q_e_ = q_max_k_Lan_C_e_/(1 + k_Lan_C_e_) (3)

The Freundlich isotherm model is described using Equation (4):

q_e_ = k_Fre_C_e_^1/n^ (4)

where C_e_ (mg/L) is the equilibrium concentration of Pb^2+^ in aqueous solutions; q_max_ (mg g^−^^1^) is the maximum monolayer capacity for Pb^2+^ uptake based using the Langmuir model; k_Lan_ (L mg^−^^1^) is the Langmuir uptake constant, which represents the bond energy; k_Fre_ (mg g^−^^1^(L/mg)^−1/n^) is the Freundlich model constant, representing the saturation adsorption capability of the adsorbent [9]; and n is the heterogeneity factor. The value of n represents the interactions between the adsorbent and the metal ion [32].

**Figure 6:**
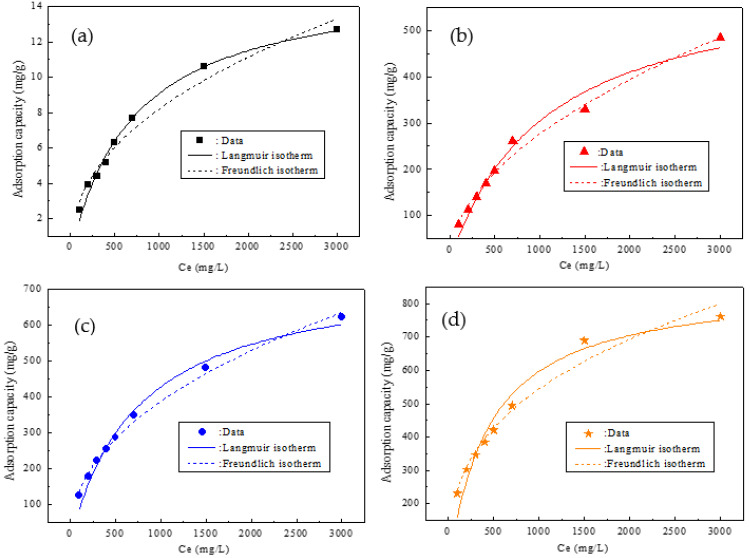
Adsorption isotherms with different concentrations of Pb^2+^ in raw steel slag and the nanocomposite samples prepared with different additions of MDP: (**a**) raw steel slag (S0), (**b**) S1 (0 g MDP), (**c**) S2 (10 g MDP), and (**d**) S3 (30 g MDP).

Figure 6 demonstrates the adsorption isotherms with different concentrations of Pb^2+^ in raw steel slag and the nanocomposite samples prepared with different additions of MDP, and the fitting parameters are shown in Table 1.

For raw steel slag (S0), the value of the correlation coefficient (R^2^) for the Langmuir model was above 0.98, and was higher than that for the Freundlich model, indicating that the Langmuir model could better describe the adsorption isotherm processes of Pb^2+^ in S0. In contrast, the R^2^ for the Freundlich model was more than 0.97, and was higher than that for the Langmuir model for all the three nanocomposite samples (S1, S2, and S3), implying that the Freundlich model could better express the adsorption isotherm processes of Pb^2+^ in S1, S2, and S3. These indicated that: the adsorption of Pb^2+^ in raw steel slag (S0) was a monolayer adsorption process, that the three nanocomposite samples possessed heterogeneous adsorption surfaces, and that the adsorption of Pb^2+^ in S1, S2, and S3 was a multilayer adsorption process [33]. This was probably due to the fact that raw steel slag (S0) had only one effective adsorption composition (Ca_2_SiO_4_), but S1 had two effective adsorption components (Ca_2_SiO_4_ and HAP), and both S2 and S3 had three effective adsorbent components (Ca_2_SiO_4_, HAP, and BCC), as shown in Figure 1. The value of n for all the four samples (S0, S1, S2, and S3) was greater than 1, suggesting high interactions between Pb^2+^ and all the four samples [32]. The numerical value of k_Fre_ (mg g^−^^1^(L/mg)^−1/n^) for S0, S1, S2, and S3 was 0.396, 8.151, 17.43, and 48.43, respectively, indicating that the formation of nanocomposites greatly improved the saturation adsorption capability of Pb^2+^, and the obtained carnation-like nanocomposite sample (S3) obtained the highest adsorption capacity. From the Langmuir model simulation, the maximum adsorption capacity (q_max_) of Pb^2+^ in S0, S1, S2, and S3 was 15.66 mg g^−^^1^, 623.7 mg g^−^^1^, 753.4 mg g^−^^1^, and 859.7 mg g^−^^1^, respectively, which was very close to the corresponding experimental value. The hierarchical carnation-like nanocomposite achieved Pb^2+^absorption rates 53 times higher than those for the raw steel slag (859.7 mg g^−^^1^ of S3 versus 15.66 mg g^−^^1^ of S0). Moreover, the comparisons of the adsorption capacities of Pb^2+^ in steel slag-based materials or HAP materials are shown in Table 2. [13,14,16,34,35] It can be observed that all the three nanocomposite samples showed remarkable Pb^2+^ adsorption capability compared with steel slag-based absorbents. The carnation-like HAP/BCC nanocomposite (S3) showed great advantages over the steel slag-based materials, with an adsorption capacity nearly parallel with that of the advanced nanoscale HAP-based materials. However, the price of the commercial nano HAP powder is about USD 50 per kilogram. Currently, raw steel slag is practically free of cost, at about USD 4 per ton (USD 0.004 per kilogram), and the other chemicals are all readily and cheaply available; therefore, the carnation-like HAP/BCC nanocomposite (S3) is the overwhelming favorite due to its low cost (currently, the cost is evaluated to be only about USD 3.5 per kilogram).

### 3.3. Magnetic Performance and Magnetic Separation

**Figure 7:**
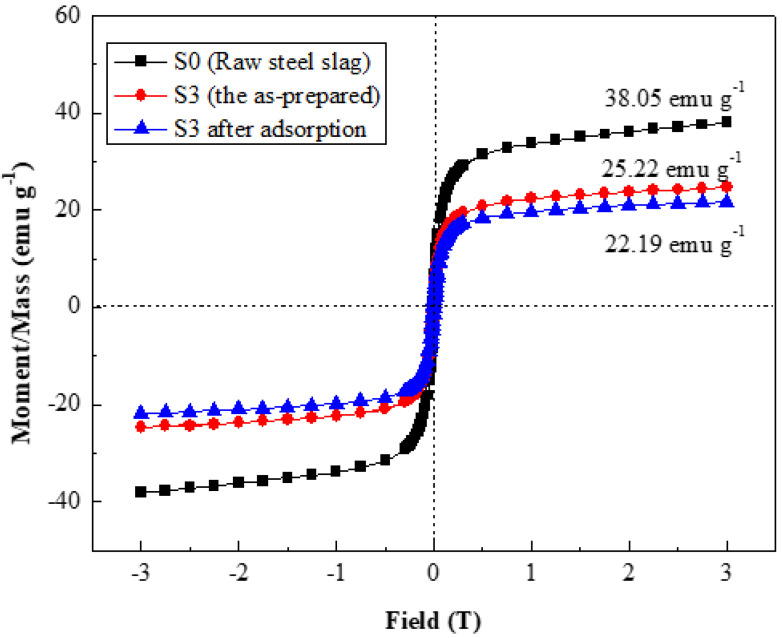
VSM curves for raw steel slag, the as-prepared S3, and S3 after adsorption of Pb^2+^.

Figure 7 illustrates the VSM curves for raw steel slag, the as-prepared S3, and S3 after the adsorption of Pb^2+^. The raw steel slag had good superparamagnetism, and the saturation magnetization reached 38 emu g^−^^1^. The saturation magnetization of the carnation-like HAP/BCC nanocomposite (S3) decreased to 25 emu g^−^^1^, and after adsorption of Pb^2+^, this slightly reduced to 22 emu g^−^^1^. The good superparamagnetism, obtained from the Fe_2.936_O_4_ of steel slag, is favorable to the magnetic separation and recovery of the absorbents. The recovery efficiency of the separated S3 was still over 95% after being magnetically separated 5 times, as shown in Table S2.

### 3.4. Formation and Adsorption Mechanism

**Figure 8:**
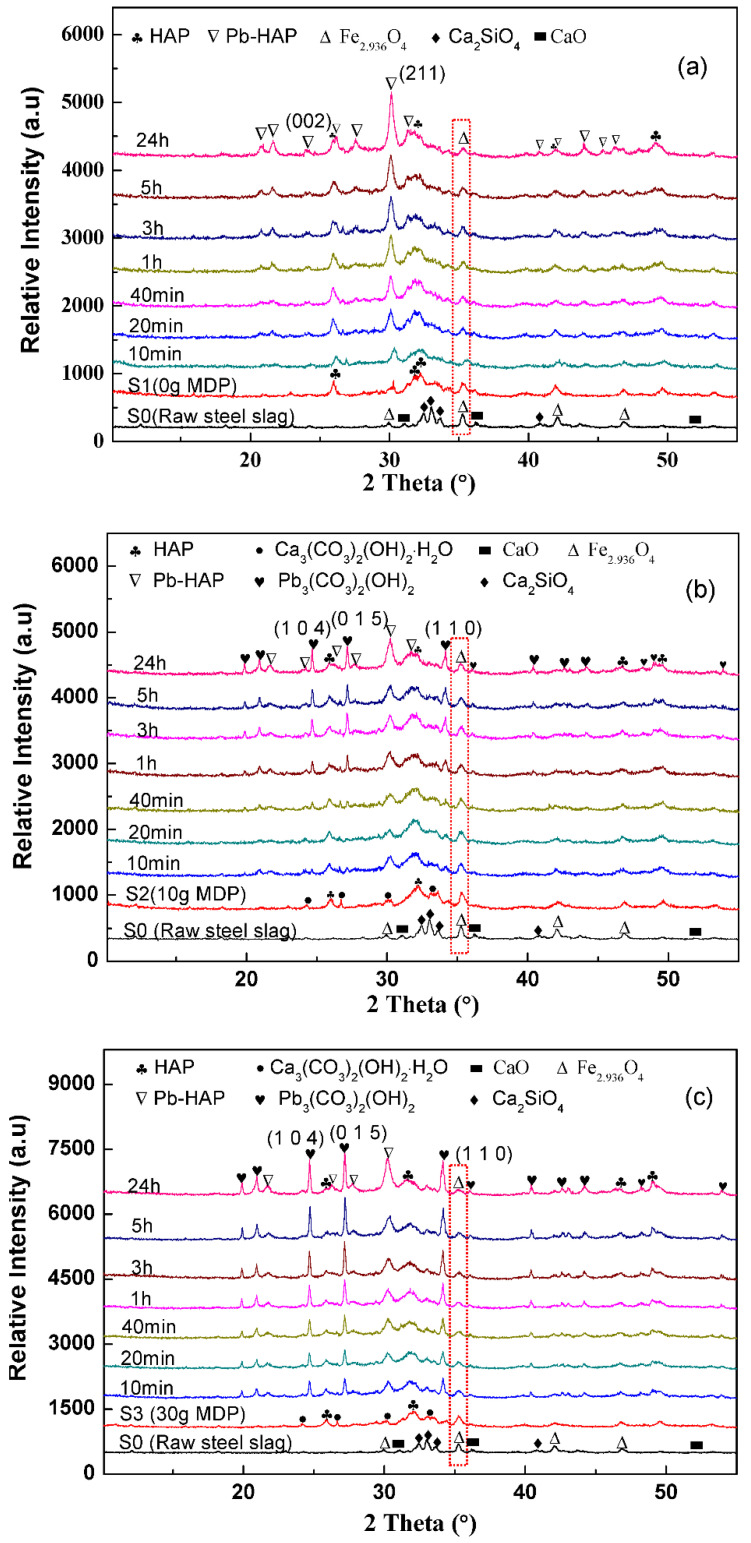
XRD patterns of raw steel slag and the three nanocomposite samples before and after adsorption at different times, with an initial Pb^2+^ concentration of 700 ppm: (**a**) S1 (0 g MDP), (**b**) S2 (10 g MDP), and (**c**) S3 (30 g MDP).

Figure 8 shows the XRD patterns for raw steel slag and the three nanocomposite samples, before and after adsorption at different times, with an initial Pb^2+^ concentration of 700 ppm (the XRD pattern results for the adsorption at 1500 ppm Pb^2+^ and 3000 ppm Pb^2+^ are shown in Figures S4–S9 in the Supplementary Materials). It can be clearly observed that: S1 had only one Pb-contained phase—Pb-HAP—and the content of Pb-HAP increased with the adsorption time; both S2 and S3 had two Pb-contained phases—Pb-HAP and Pb_3_(CO_3_)_2_(OH)_2_—and the content of the two Pb-contained phases also increased with the adsorption time, but the content of Pb_3_(CO_3_)_2_(OH)_2_ for S3 was higher than that for S2, according to the intensities of the diffraction peaks; all the samples possessed a Fe_2_._936_O_4_ phase, which was the source of superparamagnetism. These results corresponded with the results of Figure 1. In contrast to the phases in steel slag, the modified sample-S1 contained an HAP phase with a small amount of CaHPO_4_, and S2 and S3 possessed HAP and BCC, with a small amount of CaHPO_4_. The content of BCC increased with the addition of MDP: S1 had no BBS phase, and S3 achieved a higher BCC content than did S2. This was mainly due to the different additions of MDP during the preparation process (the addition of MDP for S1, S2, and S3 was 0 g, 10 g, and 30 g, respectively). There was no BCC phase for S1 because there was no addition of MDP during the preparation process. MDP (mono-dodecyl phosphate) has a phosphate group and an alkyl chain, with 12 carbon atoms. The BCC phase of S2 and S3 possibly originated from the reaction of Ca^2+^ and carbon chains during calcination in the air. After adsorption of Pb^2+^, the HAP (Ca_10_(PO_4_)_6_(OH)_2_) phase was converted to Pb-HAP (Pb_10_(PO_4_)_6_(OH)_2_), and the BCC ((Ca_3_(CO_3_)_2_(OH)_2_·H_2_O) turned into Pb_3_(CO_3_)_2_(OH)_2_. The particle sizes of S1, S2, and S3 before and after the adsorption of Pb^2+^ were calculated according to Scherrer equation, as shown in Table S3 of the Supplementary Materials. The specific surface areas of S1, S2, and S3 after the adsorption of Pb^2+^ were 25.59 m^2^ g^−1^, 38.68 m^2^ g^−1^, and 20.38 m^2^ g^−1^, respectively (see Figure S10 in the Supplementary Materials).

**Figure 9:**
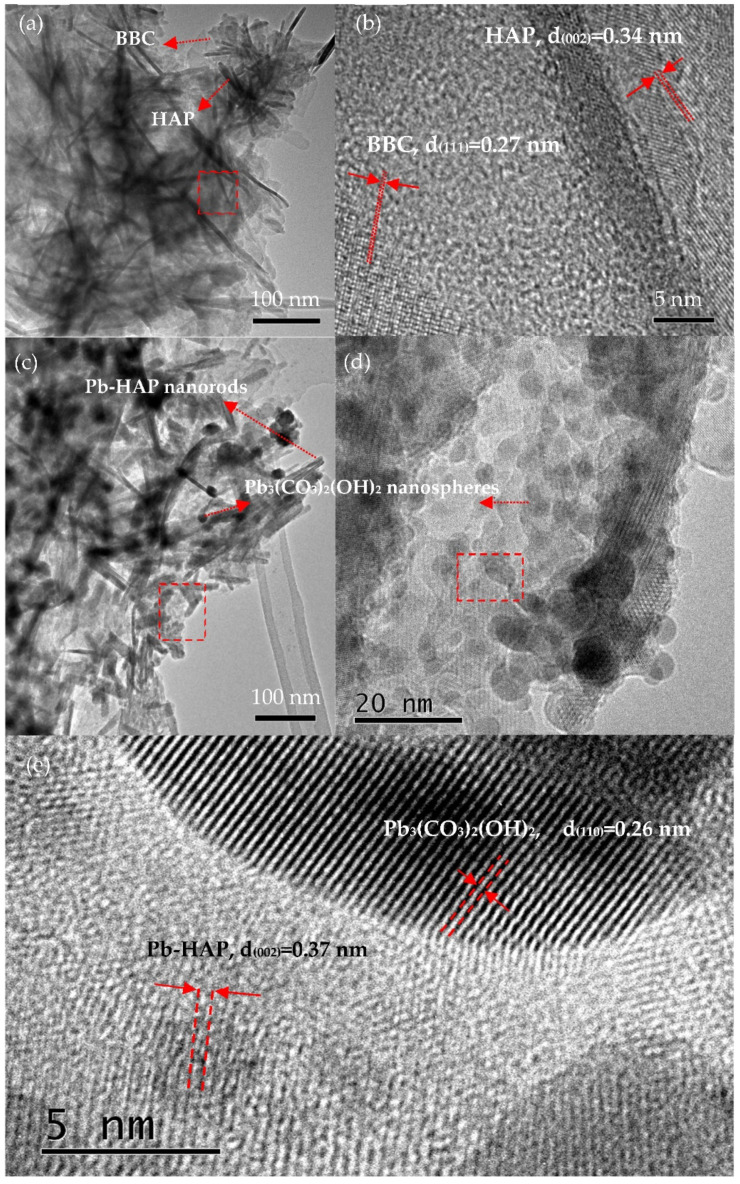
TEM and HRTEM images of S3 before (**a**,**b**) and after (**c**–**e**) adsorption of Pb^2+^.

The TEM and high resolution TEM (HRTEM) images of S3 before and after the adsorption of Pb^2+^, as shown in Figure 9, further confirmed the conversions of these phases: before adsorption, the as-prepared S3 possessed BCC nanosheets set with HAP nanoneedles (just like the petals and stems of carnation nanoflowers (Figure 2g)); then, after the adsorption of Pb^2+^, the BCC nanosheets were converted to sphere-like Pb_3_(CO_3_)_2_(OH)_2_ nanoparticles, and the HAP nanoneedles turned into HAP nanorods (as if the carnation nanoflowers bore nano-fruits).

These results were consistent with the results of the kinetic adsorption analysis, i.e., the adsorption processes were chemisorption processes. Therefore, the main effective adsorption component for S1 was the HAP phase, that of S2 and S3 was a mixture of HAP and BCC, and the Ca^2+^ of lappa-like HAP or carnation-like HAP/BCC caused ion exchange reactions with Pb^2+^ during the adsorption process. Although S3 had the relatively lowest specific surface area (46.67 m^2^ g^−^^1^) among the four samples, it obtained the greatest k_Fre_ value (48.43, which is 2.8 times that of S2, 6 times that of S1, and 122.3 times that of S0) and the highest maximum adsorption capacity of Pb^2+^ (q_max_ = 859.7 mg g^−^^1^). These results were likely due to the fact that S3 contained much more BCC, and BCC has a much lower solubility product than does HAP [21,22], causing the Ca^2+^ of BCC to more easily precipitate ion exchange reactions with Pb^2+^; meanwhile, the hierarchical carnation-like morphologies of S3 greatly increased the number of interaction sites between the Ca^2+^ of BCC, HAP, and Pb^2+^ in the solution, resulting in a huge improvement in the adsorption capacity of Pb^2+^.

**Figure 10:**
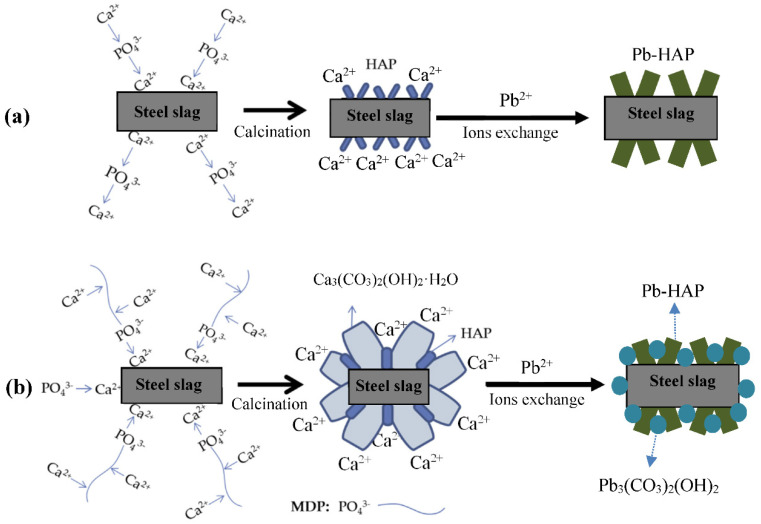
Schematic diagram of the formation mechanism and the Pb^2+^ adsorption mechanism of the hierarchical nanoflower-like nanocomposite samples: (**a**) with no addition of MDP, and (**b**) with the addition of MDP.

On the basis of the aforementioned discussion, the formation mechanism and the Pb^2+^ adsorption mechanism of the hierarchical calcium-enriched nanoflowers of modified steel slag can be summarized, as shown in Figure 10.

When no MDP was added, the phosphate ions (PO_4_^3−^) first anchored on the surface of the steel slag by interacting with the Ca^2+^ ions, and then the added Ca^2+^ ions reacted with the anchored phosphate ions and formed a crystal nucleus of HAP nanoneedles on the surfaces of steel slag particles. The subsequent HAP crystal growth was driven by an increase in Ca^2+^ and PO_4_^3−^ ions, and calcinations helped to form lappa-like HAP nanoflowers on the steel slag particles. In the aqueous solution of Pb^2+^, the Ca^2+^ ions of the lappa-like HAP nanoflower caused ion exchanges with Pb^2+^, and finally, Pb-HAP was formed. This entire process is shown as Figure 10a. Figure 10b illustrates the diagram for the samples prepared with the addition of MDP: the added MDP molecules first anchored on the surface of the steel slag through the interactions between the phosphate groups of MDP and the Ca^2+^ ions of steel slag (CaO phase). Then, the added Ca^2+^ ions interacted with the phosphate groups and the alkyl chains of the MDP molecules, and the subsequent PO_4_^3−^ ions and additional Ca^2+^ precipitated in the complex of Ca^2+^ and MDP, and finally, carnation-like HAP/BCC was formed, after calcination in the air. The complex of HAP and BCC was probably the reason for the formation of the stable BCC phase. Moreover, in the aqueous solution of Pb^2+^ (the pH is about 4.1–4.5), Pb^2+^ was the dominated species, in the pH range of 2.5–5.9 [36]; therefore, the Ca^2+^ ions of the carnation-like HAP/BCC nanoflowers precipitated ion exchanges with Pb^2+^, and Pb-HAP and Pb_3_(CO_3_)_2_(OH)_2_, respectively, were formed. When the MDP molecules were not adequate to cover the surfaces of the steel slag particles, some surface areas experienced the reaction process shown in Figure 10a, resulting in the mixture of lappa-like and carnation-like morphologies for S2.

## 4. Conclusions

In summary, nanocomposites with lappa-like HAP and/or carnation-like HAP/BCC were successfully achieved through controlling the addition of MDP during the preparation process. Without the addition of MDP, the lappa-like HAP nanocomposite was obtained, and with the addition of 30 g MDP, the carnation-like HAP/BCC nanocomposite was acquired. The pseudo-second-order and Freundlich models were more powerful than other models in expressing the kinetic and equilibrium behaviors of Pb^2+^ adsorption in the nanocomposites, indicating that the adsorptions belonged to the chemisorption and multilayer adsorption processes. The main effective adsorption components for the nanocomposite were HAP and BCC. Through exchanges between Ca^2+^ ions and Pb^2+^ ions in the solution, the HAP and BCC phases converted to Pb-HAP and Pb_3_(CO_3_)_2_(OH)_2_, respectively. The structure of the hierarchical calcium-enriched nanoflowers gave the carnation-like HAP/BCC nanocomposite a high Pb^2+^ adsorption capacity of 860 mg g^−^^1^, which was 54 times that of the raw steel slag. Additionally, the carnation-like HAP/BCC nanocomposite possessed good superparamagnetism and magnetic recovery efficiency, even after the adsorption of Pb^2+^. The low cost, significant adsorption capacity, and good magnetic recovery efficiency provides the carnation-like HAP/BCC nanocomposite with significant potential for the treatment of heavy-metal polluted wastewater and soil.

## Figures and Tables

**Table 1:** Fitting parameters for the adsorption isotherms of Pb^2+^ for different concentrations of raw steel slag and the nanocomposite samples prepared with different additions of MDP.

Sample	Langmuir Isotherm			Freundlich Isotherm		
	q_max_ (mg g^−1^)	k_Lan_ (L mg^−1^) ×10^−1^	R^2^	n	k_Fre_ (mg g^−1^(L/mg)^−1/n^)	R^2^
S0(Raw steel slag)	**15.66**	0.014	0.989	2.278	0.396	0.972
S1(0 g-MDP)	**623.7**	0.010	0.974	1.960	8.151	0.989
S2(10 g-MDP)	**753.4**	0.013	0.982	2.228	17.43	0.993
S3(30 g-MDP)	**859.7**	0.023	0.951	2.856	48.43	0.971

**Table 2:** Comparison of the adsorption capacities of Pb^2+^ on steel slag-based materials or HAP materials.

Absorbent	Surface Area (m^2^ g^−1^)	q_max_ (mg g^−1^)	References
Raw steel slag (300 mesh) (S0)	3.31	15.66	This work
Lappa-like HAP nanocomposite (S1)	52.24	623.7	This work
Mixed nanoflower-like HAP/BCC nanocomposite (S2)	78.94	753.4	This work
**Carnation-like HAP/BCC nanocomposite (S3)**	**46.67**	**859.7**	**This work**
Steel slag-derived calcium silicate hydrate	76.5	550	[13,14]
3D flower-like HAP	N	30	[34]
Bitter gourd-shaped nanoscale HAP	77.25	815	[16]
HAP-biochar nanocomposite	126.4	961.5	[35]
HAP/calcium silicate hydrate	84.54	946.7	[10]

## Data Availability

Not applicable.

## Supplementary Materials

The following supporting information can be downloaded at: https://www.mdpi.com/article/10.3390/molecules27175565/s1, Figure S1: BJH pore size distribution of raw steel slag (S0); Figure S2: The TEM-EDS mapping data of S2; Figure S3: The TEM-EDS mapping data of S3; Figure S4: XRD patterns of raw steel slag (S0) and sample S1 before and after adsorption at different times with an initial Pb^2+^ concentration of 1500 ppm; Figure S5: XRD patterns of raw steel slag (S0) and sample S1 before and after adsorption at different times with an initial Pb^2+^ concentration of 3000 ppm; Figure S6: XRD patterns of raw steel slag (S0) and sample S2 before and after adsorption at different times with an initial Pb^2+^ concentration of 1500 ppm; Figure S7: XRD patterns of raw steel slag (S0) and sample S2 before and after adsorption at different times with an initial Pb^2+^ concentration of 3000 ppm; Figure S8: XRD patterns of raw steel slag (S0) and sample S3 before and after adsorption at different times with an initial Pb^2+^ concentration of 1500 ppm; Figure S9: XRD patterns of raw steel slag (S0) and sample S3 before and after adsorption at different times with an initial Pb^2+^ concentration of 3000 ppm; Figure S10: Nitrogen adsorption/desorption isotherm curves of S1, S2, and S3 after adsorption of Pb^2+^; Figure S11: Lappa-like nanoflower morphology of S1; Figure S12: Carnation-like nanoflower morphology of S3; Table S1: The fitting parameters for the kinetic adsorption curves of Pb^2+^ with different concentrations on raw steel slag and the nanocomposite samples prepared with different additions of MDP; Table S2: The recovery efficiency of S3 by magnetic separation; Table S3: The particle size of S1, S2, and S3 before and after adsorption of Pb^2+^ was calculated according to Scherrer equation.

## Sample Availability

Samples of the compounds are not available from the authors.
