# Supplementary material for: Synthesis of a Magnetic Carnation-like Hydroxyapatite/Basic Calcium Carbonate Nanocomposite and Its Adsorption Behaviors for Lead Ions in Water
Source: Molecules. 2022 Aug 29;27(17):5565. doi: 10.3390/molecules27175565 (PMC9457816; doi:10.3390/molecules27175565)
Supplement: Supplementary file 1 [file molecules-27-05565-s001.zip › molecules-1879838-supplementary.pdf]

molecules

Supplementary Materials

Synthesis of a Magnetic Carnation-like Hydroxyapatite/Basic Calcium Carbonate Nanocomposite and Its Adsorption Behaviors for Lead Ions in Water

Haifeng Guo [1,*], Siru Hu [1], Zongli Wang [1], Yutong Li [2], Xinshuang Guo [1,*], Ziling He [1], Wenbin Wang [1], Jun Feng [1], Kangyun Yang [1] and Hong Zheng [1]

[1] Engineering & Technology Research Center for Environmental Protection Materials and Equipment of Jiangxi Province, College of Materials and Chemical Engineering, Pingxiang University, Pingxiang 337055, China
[2] School of Statistics and Data Science, Nankai University, Tianjin 300071, China
* Correspondence: guohaifeng720@163.com or 21010039@pxu.edu.cn (H.G.); xsguo12b@alum.imr.ac.cn (X.G.)

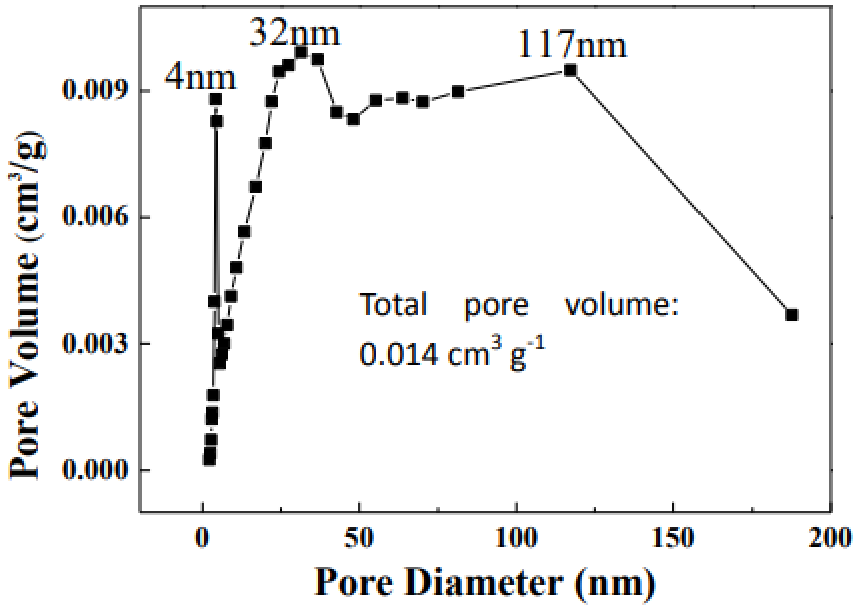

Figure S1. BJH pore size distribution of raw steel slag (S0).

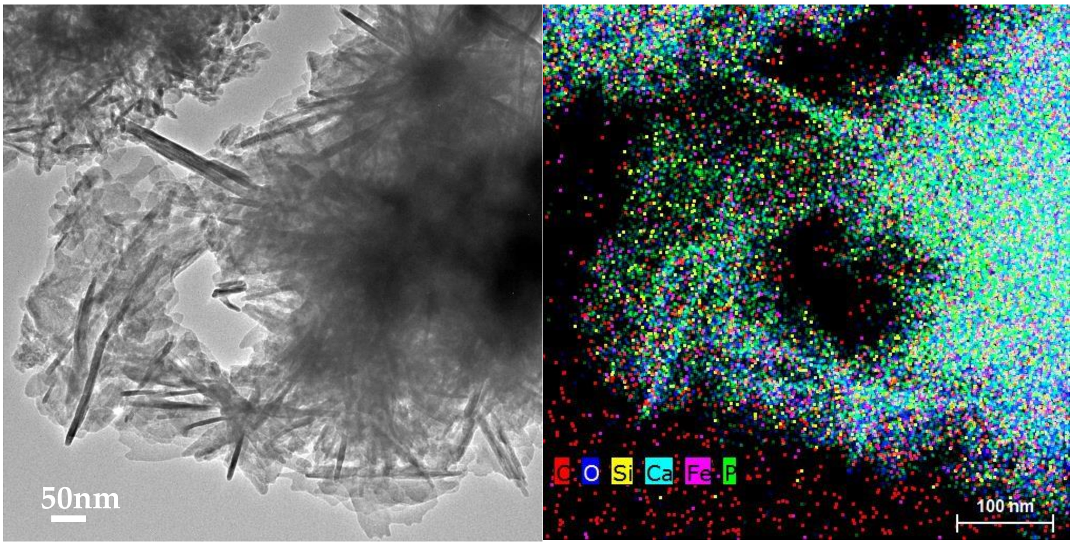

Spectrum: Map.xls

El	AN	Series	unn. C [wt.%]	norm. C [wt.%]	Atom. C [at.%]	Error (1 Sigma) [wt.%]
Ca	20	K-series	50.75	50.75	33.74	1.57
O	8	K-series	20.50	20.50	34.14	0.67
P	15	K-series	17.48	17.48	15.03	0.57
**C**	**6**	**K-series**	**6.07**	**6.07**	**13.48**	**0.25**
Fe	26	K-series	2.81	2.81	1.34	0.13
Si	14	K-series	2.39	2.39	2.27	0.07
		Total:	100.00	100.00	100.00	

Figure S2. The TEM-EDS mapping data of S2.

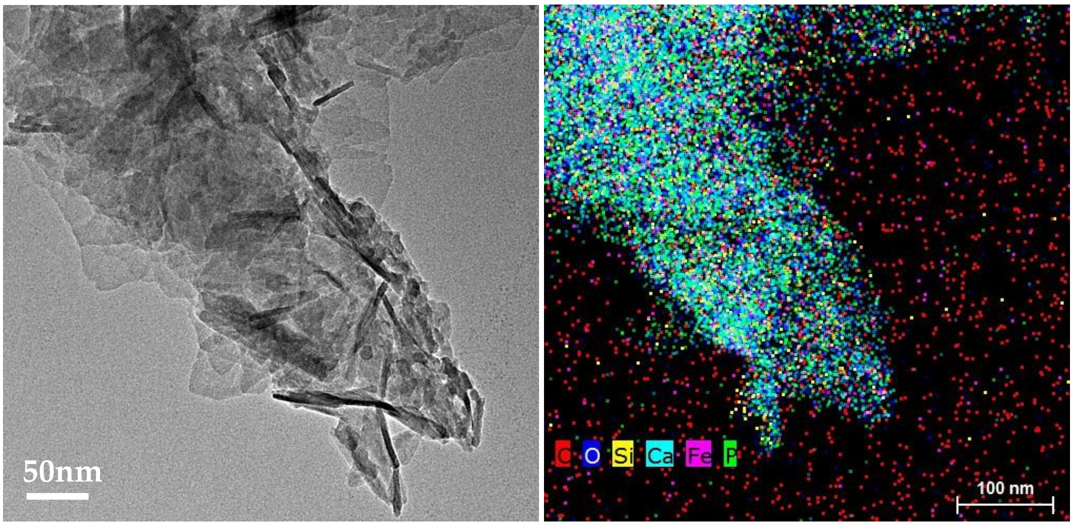

Spectrum: Map.xls

El	AN	Series	unn. C [wt.%]	norm. C [wt.%]	Atom. C [at.%]	Error (1 Sigma) [wt.%]
Ca	20	K-series	36.76	36.76	19.38	1.19
O	8	K-series	29.40	29.40	38.82	0.99
**C**	**6**	**K-series**	**17.69**	**17.69**	**31.11**	**0.70**
P	15	K-series	13.36	13.36	9.11	0.48
Si	14	K-series	1.43	1.43	1.08	0.09
Fe	26	K-series	1.35	1.35	0.51	0.11
		Total:	100.00	100.00	100.00	

Figure S3. The TEM-EDS mapping data of S3.

The carbon atom content of S2 is 13.48 % and the carbon atom content of S3 is 31.11 %, indicating that S3 has a more content of BCC ($Ca_3(CO_3)_2(OH)_2\bullet H_2O$) than S2 does (about 2.31 times). The phosphorus atom content of S2 is 15.03 % and the phosphorus atom content of S3 is 9.11 %, suggesting that S2 has a more content of HAP ($Ca_{10}(PO_4)_6(OH)_2$) than S3 does (about 1.65 times).

Table S1. The fitting parameters for the kinetic adsorption curves of Pb^{2+} with different concentrations on raw steel slag and the nanocomposite samples prepared with different addition of MDP.

Sample	Pb^{2+} concentration ($mg \cdot L^{-1}$)	Pseudo-first-order model			Pseudo-second-order model		
		q_e ($mg \cdot g^{-1}$)	k_1 (min^{-1})	R^2	q_e ($mg\ g^{-1}$)	$k_2 \times 10^{-1}$ ($(g(mg \cdot min)^{-1}$)	R^2
S0(Raw steel slag)	700	11.52	0.0069	0.989	12.78	0.008	0.996
	1500	12.32	0.0082	0.963	13.34	0.011	0.989
	3000	13.95	0.0129	0.967	15.30	0.012	0.995
S1(0g-MDP)	700	334.4	0.0054	0.972	364.0	0.0003	0.981
	1500	402.1	0.0067	0.972	438.5	0.0003	0.990
	3000	567.4	0.0066	0.991	641.5	0.0001	0.992
S2(10g-MDP)	700	342.2	0.0604	0.893	360.4	0.0030	0.974
	1500	484.7	0.0273	0.917	505.8	0.0011	0.970
	3000	625.0	0.0310	0.905	651.9	0.0010	0.970
S3(30g-MDP)	700	496.6	0.0465	0.914	521.5	0.0002	0.980
	1500	702.6	0.0296	0.945	740.4	0.0007	0.985
	3000	737.0	0.0765	0.910	778.3	0.0017	0.981

Table S2. The recovery efficiency of S3 by magnetic separation.

Repeat times	Before adsorption		Before adsorption of Pb^{2+}	
	The quality before magnetic separation (g)	Magnetic recovery efficiency (%)	The quality before magnetic separation (g)	Magnetic recovery efficiency (%)
Initial	0.500	100	0.513	100
1	0.497	99.46	0.510	99.36
2	0.496	99.1	0.501	97.68
3	0.491	98.14	0.499	97.33
4	0.484	96.84	0.492	95.98
5	0.482	96.36	0.491	95.63

Note: the powders were added into a 50ml PP plastic tube, and separated by a neodymium magnet with a remanence of about 1.2 T.

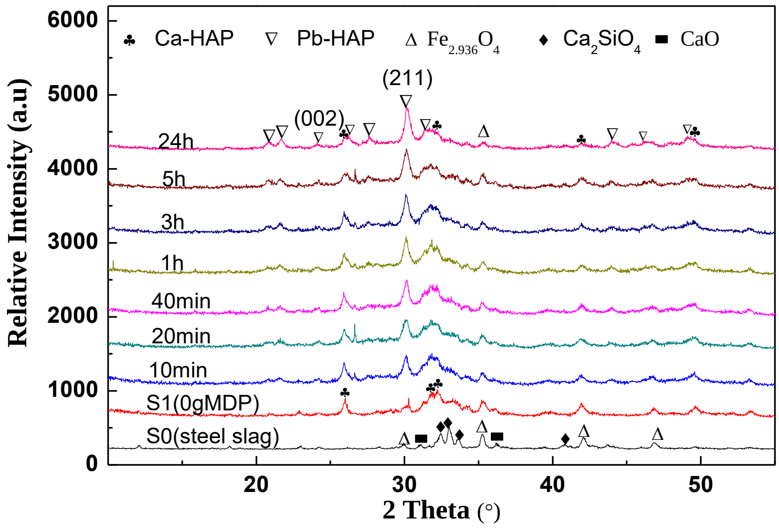

Figure S4. XRD patterns of raw steel slag (S0) and sample S1 before and after adsorption at different time with an initial Pb^{2+} concentration of 1500 ppm.

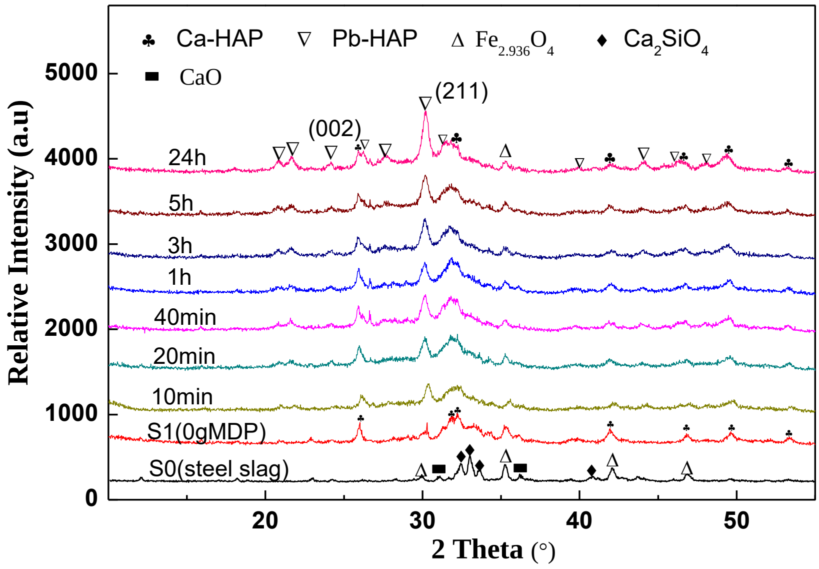

Figure S5. XRD patterns of raw steel slag (S0) and sample S1 before and after adsorption at different time with an initial Pb^{2+} concentration of 3000 ppm.

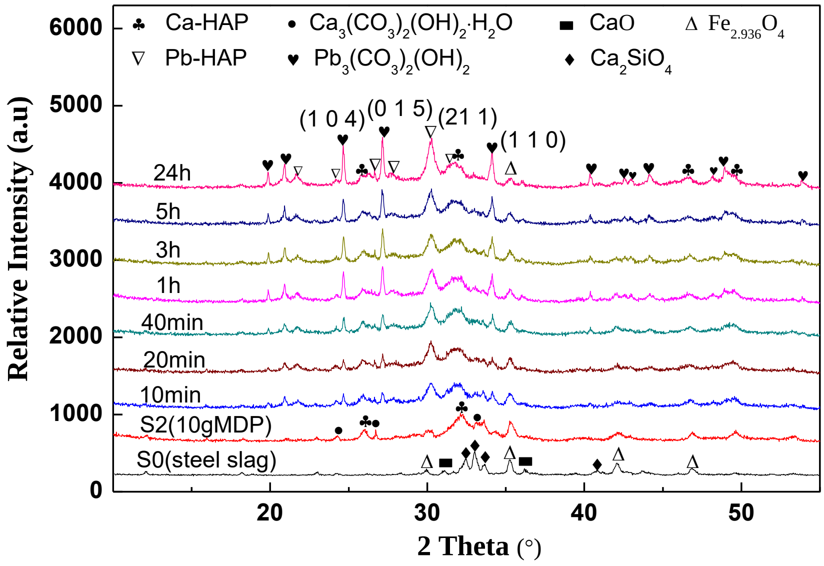

Figure S6. XRD patterns of raw steel slag (S0) and sample S2 before and after adsorption at different time with an initial Pb^{2+} concentration of 1500 ppm.

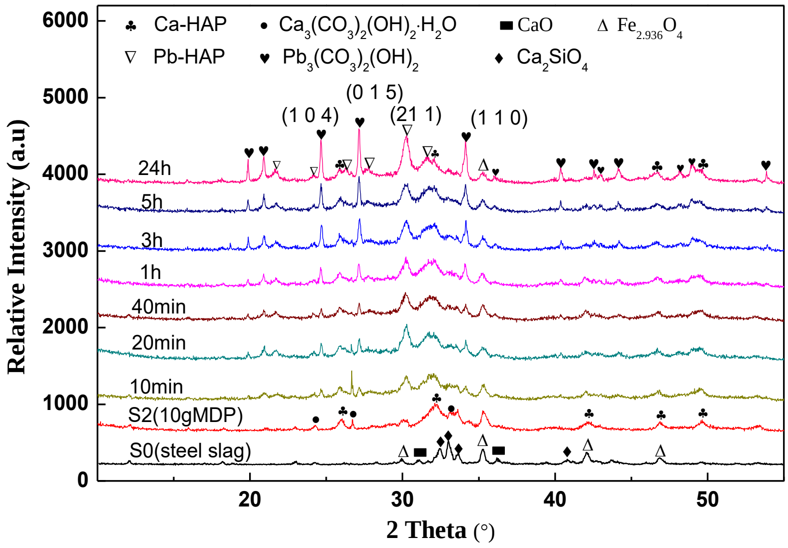

Figure S7. XRD patterns of raw steel slag (S0) and sample S2 before and after adsorption at different time with an initial Pb^{2+} concentration of 3000 ppm.

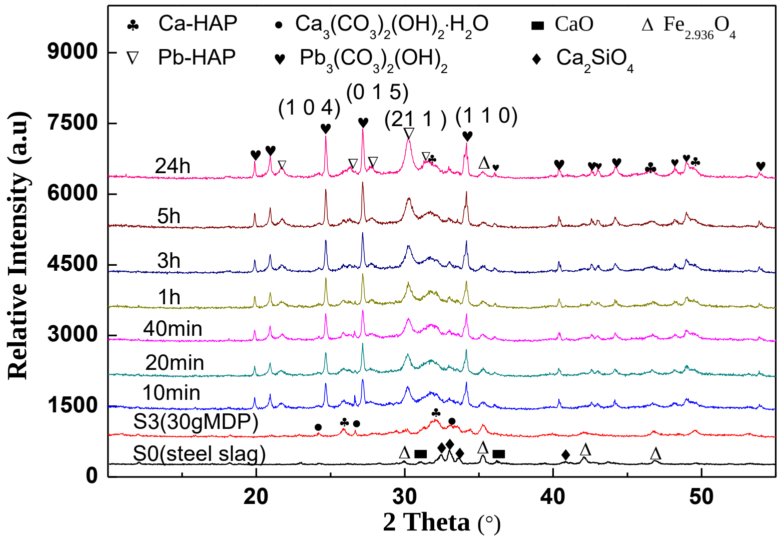

Figure S8. XRD patterns of raw steel slag (S0) and sample S3 before and after adsorption at different time with an initial Pb^{2+} concentration of 1500 ppm.

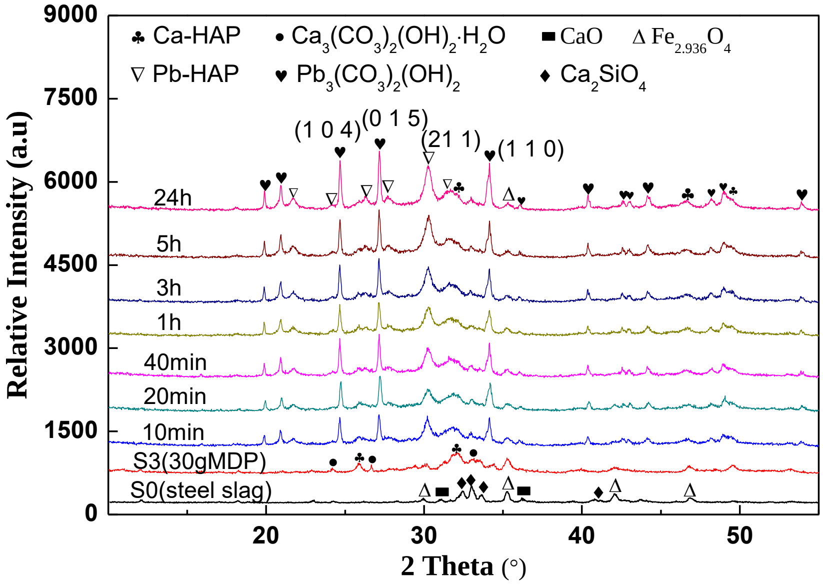

Figure S9. XRD patterns of raw steel slag (S0) and sample S3 before and after adsorption at different time with an initial Pb^{2+} concentration of 3000 ppm.

Table S3. The particle size of S1, S2 and S3 before and after adsorption of Pb^{2+} calculated according to Scherrer equation. For S1: the particle size decreased from 25.2 nm to 20.6 nm after adsorption of Pb^{2+} (HAP to Pb-HAP); for S2: the particle size increased from 23.0 nm to 34.9 nm after adsorption of Pb^{2+} (HAP to Pb-HAP), and slightly decreased from 26.3 nm to 23.5 nm (BCC to $Pb_3(CO_3)_2(OH)_2$); and for S3: the particle size increased from 34.5 nm to 37.8 nm after adsorption of Pb^{2+} (HAP to Pb-HAP), and decreased from 35.1 nm to 21.2 nm (BCC to $Pb_3(CO_3)_2(OH)_2$).

Sample	FWHM (°)	Peak position (°)	Crystal plane direction	Crystallite size (nm)
S1 before adsorption	0.323	25.99	(002) of HAP	25.2
S1 after adsorption	0.394	24.02	(002) of Pb-HAP	20.6
S2 before adsorption	0.354	25.89	(002) of HAP	23.0
	0.315	33.05	(111) of BCC	26.3
S2 after adsorption	0.233	24.07	(002) of Pb-HAP	34.9
	0.354	35.29	(110) of $Pb_3(CO_3)_2(OH)_2$	23.5
S3 before adsorption	0.236	25.90	(002) of HAP	34.5
	0.236	33.06	(111) of BCC	35.1
S3 after adsorption	0.215	24.09	(002) of Pb-HAP	37.8
	0.394	35.32	(110) of $Pb_3(CO_3)_2(OH)_2$	21.2

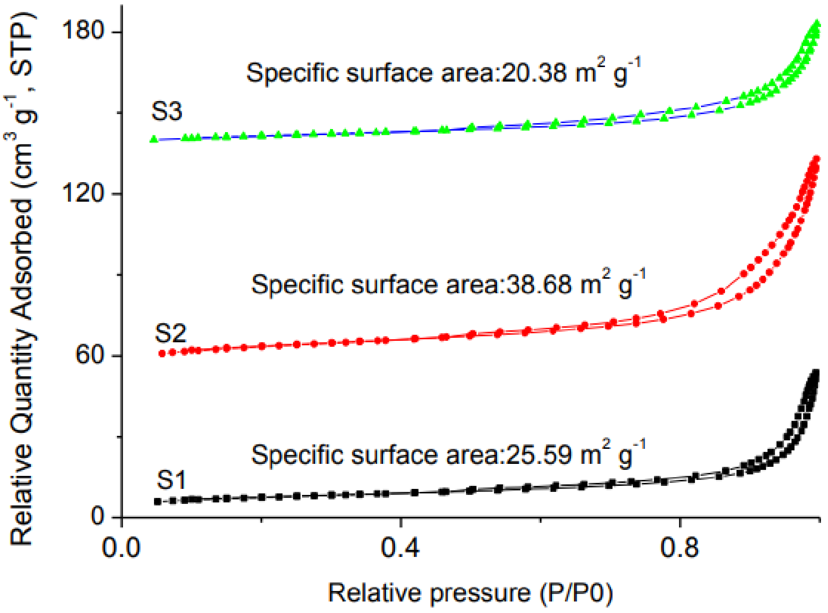

Figure S10. Nitrogen adsorption/desorption isotherm curves of S1, S2 and S3 after adsorption of Pb^{2+}. STP is standard temperature and pressure.

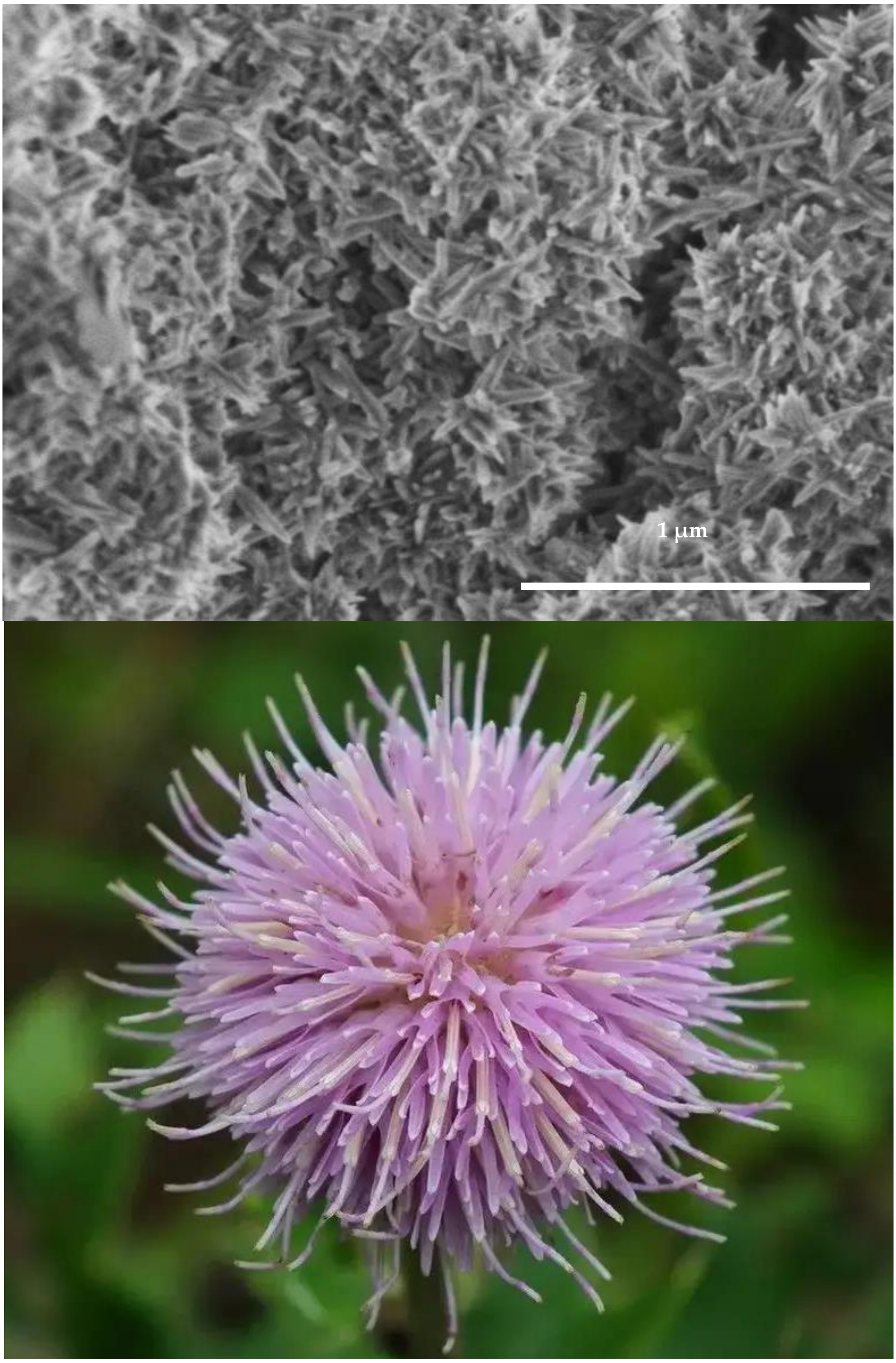

Figure S11. Lappa-like nanoflower morphology of S1.

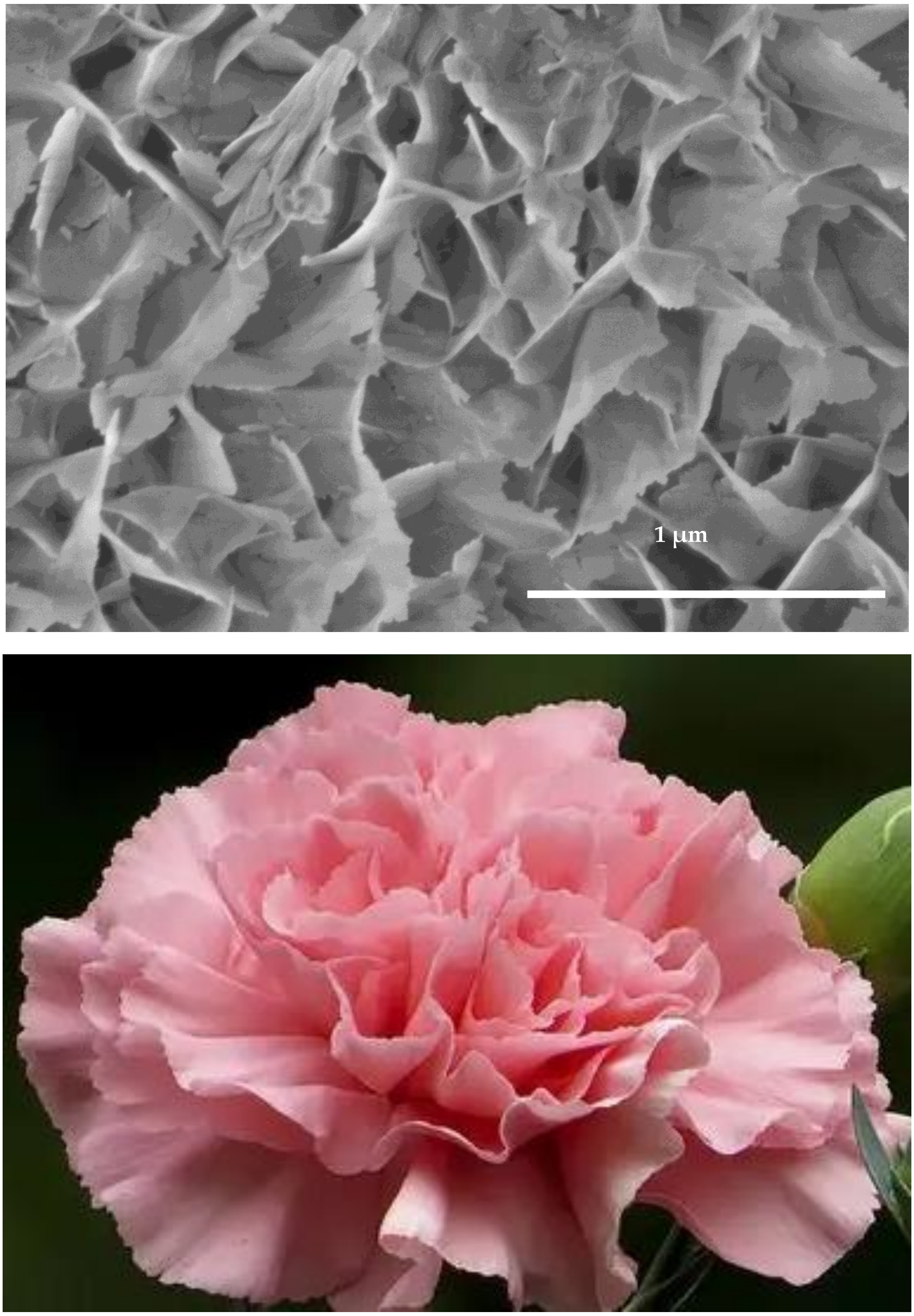

Figure S12. Carnation-like nanoflower morphology of S3.
